# Adaptation to Delayed Force Perturbations in Reaching Movements

**DOI:** 10.1371/journal.pone.0012128

**Published:** 2010-08-11

**Authors:** Noa Levy, Assaf Pressman, Ferdinando A. Mussa-Ivaldi, Amir Karniel

**Affiliations:** 1 Department of Biomedical Engineering, Ben-Gurion University of the Negev, Beer Sheva, Israel; 2 Sensory Motor Performance Program, Rehabilitation Institute of Chicago, Chicago, Illinois, United States of America; The University of Western Ontario, Canada

## Abstract

Adaptation to deterministic force perturbations during reaching movements was extensively studied in the last few decades. Here, we use this methodology to explore the ability of the brain to adapt to a delayed velocity-dependent force field. Two groups of subjects preformed a standard reaching experiment under a velocity dependent force field. The force was either immediately proportional to the current velocity (Control) or lagged it by 50 ms (Test). The results demonstrate clear adaptation to the delayed force perturbations. Deviations from a straight line during catch trials were shifted in time compared to post-adaptation to a non-delayed velocity dependent field (Control), indicating expectation to the delayed force field. Adaptation to force fields is considered to be a process in which the motor system predicts the forces to be expected based on the state that a limb will assume in response to motor commands. This study demonstrates for the first time that the temporal window of this prediction needs not to be fixed. This is relevant to the ability of the adaptive mechanisms to compensate for variability in the transmission of information across the sensory-motor system.

## Introduction

Fast reaching movements are ballistic, voluntary movements of the arm from a starting point to a given target [1]–[2]. They last a few hundred milliseconds, and visual feedback is not operational, at least during the initial part of these movements.

Several studies suggest that the brain constructs internal models of arm dynamics to generate the motor commands needed to drive the hand along a planned trajectory. Reaching movements were most instrumental in uncovering the structure of these internal representations [3]–[8].

The normal unperturbed trajectory of the hand reaching for a target is typically a straight path from the initial position of the hand to the target, transverse with a smooth bell-shaped speed profile [1]–[2]. It has been suggested that this straight trajectory is generated by an internal model that calculates motor commands, which appropriately compensate the arm dynamics. When perturbing forces are unexpectedly applied to the hand, the motor commands are insufficient to compensate for them, and the trajectory of the hand initially deviates from this straight line. After prolonged exposure to deterministic perturbation forces, which depend on the state of motion of the hand – i.e., on its position and velocity – the internal model adapts to fit the combination of the arm dynamics and the applied force field [4], [9]. At this point, an unexpected removal of the perturbation (called a “catch-trial”) results in an erroneous movement, which generally resembles the mirror image of the initial deviation, caused by the perturbing force. This typical response to a catch trial is known as *after-effect of adaptation*.

The slow transmission rate of information in the nervous system introduces significant delays in the sensory motor loop which must be accounted for by the brain. Since the brain must also adapt to changes in these delays it is reasonable to assume that the brain needs to be able to compensate for additional external delays in the sensory motor loop. We have recently studied the effect of feedback delays on the perception of stiffness [10]–[11], aiming at understanding the capabilities of the brain in handling delay between force and position. Small delays of up to 60msec affected subjects' estimation of stiffness in a systematic way (overestimation of the surface's stiffness), but larger delays tended to disrupt the ability of subjects to discriminate stiffness. However, clear evidence of adaptation to visual feedback delays was shown in a tracking task with a delayed visual feedback [12]. Cunningham et al studied temporal delays adaptation using a driving task in a simulated environment [13], and showed that the improvement during training was a result of temporal visuo-motor adaptation.

Delayed visual feedback during reaching movements were studied by Kitazawa et al [14] who provided delayed knowledge of results, and analyzed the influence of the delay on the learning. Smith and Bowen [15] studied the effects of delayed vision during the movement and demonstrated adaptation and after-effects of learning. However, adaptation to delayed force perturbations during reaching movements has not yet been studied. It is important to note that a-priori it is not possible to generalize results concerning adaptation to visuo-motor perturbations and extend them to adaptation to force perturbations. It was suggested that these two kinds of perturbations are compensated in different ways and may employ different neural structures [16]–[17]. Altogether, while there are similarities in adaptation studies to visuomotor and force perturbations, these two processes do not always share the same neural mechanism and functional performance.

In the context of adaptation to force perturbations, although many types of force perturbations were explored, the ability of the brain to compensate delayed deterministic forces has not yet been studied. We consider here the basic question of “Can the brain adapt to delayed velocity-dependent force perturbations?”. We explored this question by exposing two groups of subjects to delayed and non-delayed velocity dependent force perturbations and observing their behavior in catch trials.

## Methods

### Experiment setup & protocol

**Figure 1:**
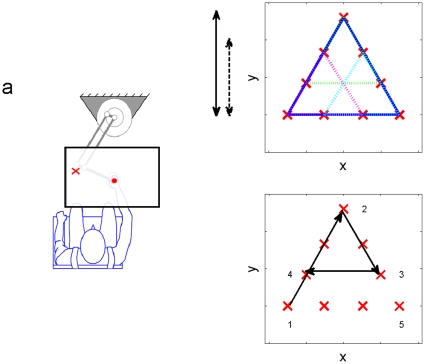
System setup and target locations. a) System Setup. The red cross is the target and the red dot is the cursor representing the hand position. b) Targets setup, the short and long arrows (dashed and sloid) show the length of the short (10 cm) and long motions (15 cm), accordingly. c) The arrows point to a typical 4 target sequence starting at target 1. Once reaching a target a limited selection was avilable for the next one. For instance, from target number 3 only target 2 or 4 would have been valid (in the currnet case, target 4 was reached).

Subjects were asked to reach several target locations with their dominant hand while holding the handle of a robotic manipulandum that could apply programmed forces. This device is a two degrees of freedom actuated mechanism (movements are restricted to the horizontal plane). Targets and feedback of hand position were presented by overhead projection. The subjects looked down on a horizontal board located above the handle, on which the location of the hand and the target were displayed. The robotic manipulandum applied programmed forces to the subject's hand, and the hand's trajectory was sampled at rate of 100 samples per second. The experimental setup is shown in Figure 1a.

The experimental setup consisted of nine static targets within a two-dimensional space, arranged as illustrated in Figure 1b (the red crosses). During an experimental session, at any given moment only one target was visible (Figure 1a). The subject moved the handle toward the displayed target. The handle location was displayed by the projector as a cursor on the screen (The subjects arm was concealed by the screen). Distance between targets was either 10 cm (short) or 15 (long) cm (Figure 1b). There were 24 possible movements: 6×3 = 18 short (each line in both directions) and 6 long (all possible movements between the 3 vertices of the big triangle). The targets were sequenced as a pseudorandom walk about the nodes of the pattern. The sequence was designed to comply with the different tasks of the experiment, as detailed below. Once reaching a specific target, subjects had a limited number of targets which they might have been asked to reach. More specifically they had either two or four targets, which depended on the actual target and if it is a vertex or not. Figure 1.c shows a typical reaching sequence between four targets as shown by arrows. Here the first reach from target 1 to 2 is a long reach. While on target 2 there are four options for a reach, two short (targets 3 or 4) and two long (targets 1 and 5). Subject was asked to reach target 3.While at target 3, the subject could have been asked to either reach back targets 2 or 4 (at the presented sequence target number 4 was reached). No long movements are available from target 3. The reaching sequence was predetermined prior to the experiment. When a target was reached, an exploding sound was played, the target vanished, and 750ms later a new target appeared. The end of movement was identified by the following condition: the hand reached a point within the radius of 0.8 cm from the target, and concurrently the velocity dropped below 5cm/sec (movement starts when the hand leaves 1cm radius from the target). The desired time for each movement, not including reaction time (i.e. the duration from leaving a target to reaching the next target), was up to 450 ms. If the subject was too slow in a specific trial, i.e. the movement was not completed within 450 ms – this trial was considered as a miss. When such a miss occurred, the target changed color from red to yellow and a short beep sound was produced (instead of the explosion sound), after which the session continued regularly. The subjects were instructed to complete each session of the experiment with the minimal possible number of misses. The experiment consisted of seven equally long (100 targets each) sessions, with short rests (about 1 minute) in between:

Null session (1): Introduction of the system and basic practice of all possible movements (short and long) with no external force. The ratio of short to long movements' incidence is approximately 3∶1. Toward the end of this session, the subjects' movements are expected to be smooth, approximately straight target-to-target trajectories, with a bell-shaped velocity profile.

Baseline session (2): All possible movements usually with no force (as in session 1). A perturbation (according to the field described below) is applied on some random scattered trials (19%).

Training sessions (3–5): Short movements with force perturbations. Each of these three sessions takes place in one of the three regions in the workspace (one of the three small triangles seen on Figure 1b at different colors). During all three sessions the force field is turned on during the movement. Exactly 10% of the movements in this stage serve as catch-trials – trials in which the force field is turned off. Catch-trials appear at random times. Catch trials as well as short and long movement were selected to obtain uniform distribution of the movements' direction.

Test sessions (6–7): All possible movements. Short movements are perturbed (similar to sessions 3–5) and long movements, which are approximately 10%, are not perturbed (long catch-trials).

Table 1 summarizes the movements in each session.

**Table 1:** Movements' specification.

Session Movement	Null	Baseline	Training	Test
Short Force	0	19	270	182
Short No Force	79	59	30	0
Long Force	0	0	0	0
Long No Force	21	22	0	18
Total	100	100	300	200

The configuration of three small triangles confined in one big triangle was chosen in order to explore the generalization of the learning which is performed for short movement to execution of long movements.

The magnitude of the force was proportional to the handle's tangential velocity with a factor of 15 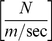. The direction of the force was clockwise normal to the velocity direction (as in [8]).

For the test group the delay was set to 50 ms (τ = 50 ms, Equation 1) and for the control group it was set to zero (τ = 0, Equation 1)
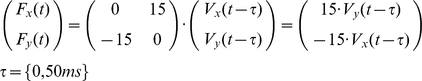
(1)The force is in units of Newtons (N), the viscosity matrix in units of 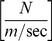, and the velocity is in units of 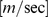.

### Subjects

Twelve subjects (age 18–33) with no known neuromotor disorders have participated in the study. They were divided into two groups: eight in the test group (delayed force field) and four in the control group (non-delayed force field). This study was approved by Northwestern's Institutional Review Board and all subjects signed the stipulated informed consent form.

Catch-trials of sessions 3–5 (training) serve as an indication of learning, providing knowledge about the type of forces which were expected by the subject. Long movements of sessions 6, 7 (test) also serve as catch-trials, since they are always performed between perturbed short movements, without any applied force. We used these trajectories to assess generalization, if any (to explore whether delay was expected also in longer movement and whether it was scaled with the length of the movement). It had been demonstrated that catch-trials interfere with learning [18]. Therefore, in any analysis made on regular trials, catch-trials and the trials immediately following them were excluded.

Two measures were used to analyze the data, the Perpendicular Distance (PD) and the Deviation Start (DS) point. These where estimated for each trajectory.

The PD is defined as the Euclidean distances between each point of the actual trajectory and the straight line connecting the start and end point of that trajectory. The Maximal Perpendicular Distance (MPD) is the greatest PD of a trajectory. This measure includes the movement's corrections, thus reflects also feedback effect.

The DS point was defined as the point in a trajectory where the deviation from a straight line first reaches 20% of the MPD of the specific trajectory. This measure was useful mainly for analyzing catch-trials: it indicates approximately the point at which the subject started to apply force perpendicular to the direction of movement, implying where and when he/she expected the external force to appear. This measure is not sensitive to differences between short and long movements, since it is obtained according to the MPD of the particular trajectory.

## Results

**Figure 2:**
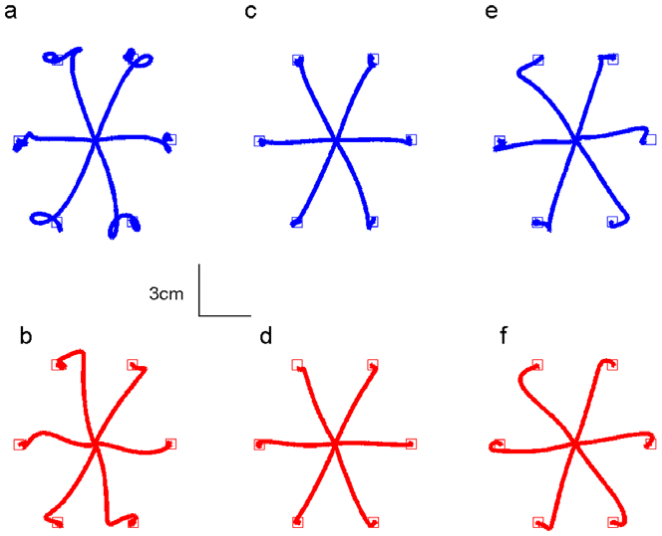
Single subject reaching trajectories. Reaching of typical subjects from both Test group (top row) and Control group (bottom row). From left to right the pre-exposure, baseline and catch trials are shown. It is evident that the test group corrects later throughout the motion than the control group. Shown is the average of all movements on each block for the pre-exposure and catch trials and the average of the last 8 movements of the baseline.

**Figure 3:**
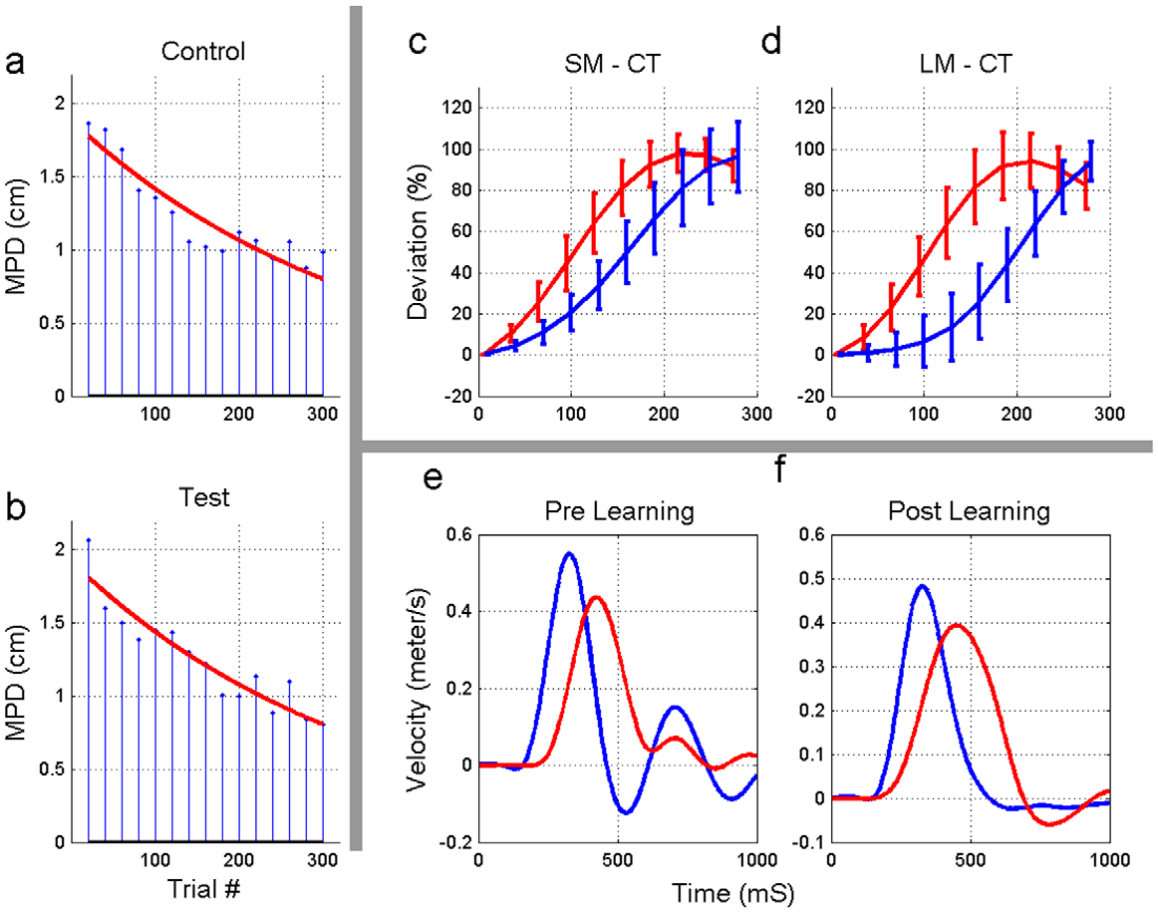
Adaptation to delayed force perturbations. (a,b) Maximal Perpendicular Distance in training sessions, not including catch and after-catch trials; the slant line is an exponential fit to the data. (c,d) The mean PD of the last catch trials (CT) of a typical subject in Test (blue) and Control (red) during training sessions (sessions 3–5). Data presented is PD normalized by movement length for short (SM) and long (LM) movements. Error bars show a single standard deviation of the mean. Note that the graphs are truncated at t = 300ms, as the post-correction part of the movement is not relevant for analysis of deviation start point. (e,f) Typical velocity profiles for Test and Control groups during pre training and catch trials. Color code is the same as above.

Figure 2 shows short reaching motion of two subjects, one from each group to all six directions practiced. It is evident that both subjects show the typical pre exposure trajectories (2, a,b), learning, in which the movement becomes straighter (2, c,d), and the after effect, where the force field is suddenly removed (2, e,f). There is a slight difference between the control and test group however the main features of adaptation and after effects of learning are clearly similarly evident in both groups. Typical velocity profiles for the two groups are shown in Figure 3 e,f. It is evident that after training subjects show a better control of their arm during movement as seen by the smaller number of oscillations.

The presence of adaptation to the delayed force field is seen clearly by analyzing the short movements of the training sessions: there is a process of learning during sessions 3–5 (declining error), which is also highlighted by after-effect in catch-trials at the end of these sessions (Figure 2). Figure 3 a,b illustrates the decreasing error using the MPD error metric. Each stem represents the average measure of 20 consequent movements, excluding catch-trials and after-catch trials, as explained in the method section. Both test and control groups show decrease in this error, indicating learning of and adaptation to the force field. In order to quantify the learning we fit an exponential function to the data of each subject.

The deviation from a straight line in catch trials starts significantly earlier in the control group compared to the test group (see Figure 3 c,d), for both short and long movements. This is consistent with the test subjects compensating a perturbation that is expected to arrive with a delay over the current velocity. In order to illustrate both short and long movement on the same Figure, the PD is normalized by the trajectory length in Figure 3 c,d For each movement direction we conducted a t-test between the test and control groups, considering the time of deviation start in short catch-trials. The resulting p-values were all smaller than 0.01. The durations of short catch-trials were significantly larger in the test group with average of 600msec compared to 530msec in the control group. This is in spite of the fact that the “instructed” duration was equal for the two groups (450msec). However, the average maximum speed during the reaching motion, for each of the groups, the control and delay, was not significantly different, indicating that the change in duration was due to the difficulty to quickly and accurately stop and not due to overall reduction of speed in the delay condition. We were unable to derive clear detailed conclusions as to the specific nature of generalization. However, it is clear that the adaptation is generalized from short to long movements in both test and control conditions and it is clear that the adaptation to the delayed force is significantly different than the adaptation to the non-delayed force field.

## Discussion

A process of learning and adaptation was clearly identified in the delayed force task. The error during the learning phase decreased with a learning rate similar to that of the control group, and there were obvious aftereffects both in late training and in test sessions of the experiment. Moreover the deviations from a straight line in catch trials were temporally shifted between the test and control trials in approximately 50ms. Therefore we can conclude that subjects successfully adapted to the 50 ms delayed velocity-dependent force field significantly differently than for the non-delayed velocity dependent force field.

Examination of catch-trials showed that the onset time of deviation differed significantly between short and long movements in the Test group subjects. This was in contrast with the behavior of the Control group, which is inconsistent with the model based on explicit representation of the delay. We have conducted further analysis attempting to test for state representation however the results were not conclusive as there was a significant change in movement duration between long and short movement. Therefore further study is required to unravel the way by which the nervous system represents the presence of delays in the state-force relationship associated with the interaction with the environment.

Studies of the neural correlates to motor adaptation suggest that certain areas at the cerebellum as well as the motor cortex demonstrate plasticity during adaptation to force perturbations [17], [19] and are probably also active during the task reported in this study. However since this study is only behavioral we can only speculate about the specific neural circuit responsible for the results reported herein. Nevertheless, we can reject the possibility of pure impedance control by co-contraction. Instead, the delay-specific after-effects support the alternative hypothesis that the adaptive control system operates by forming an internal representation of the delayed forces.

There are a few types of computational models which can account for adaptation to force perturbations, which include signal adaptation [20]–[21] or more elaborate internal models [3]–[5]. Our results demonstrate that in any such internal representation, one needs to incorporate the possibility to account for delayed force perturbations. This significantly narrows down the possible structure of this internal representation mechanism, as in other studies it was shown that such internal representation mechanisms do not include a capability to employ time representation [8], [22].

Mapping the capabilities of the motor system to adapt in face of various visuo-motor and force perturbations provides useful constraints for future theories of motor learning. In this study we provided such useful constraint by demonstrating the ability to adapt to delayed velocity dependent force perturbations.

## References

[1] Morasso P (1981). Spatial control of arm movements.. Exp Brain Res.

[2] Flash T, Hogan N (1985). The coordination of arm movements: an experimentally confirmed mathematical model.. J Neurosci.

[3] Bhushan N, Shadmehr R (1999). Computational nature of human adaptive control during learning of reaching movements in force fields.. Biol Cybernetics.

[4] Kawato M (1999). Internal models for motor control and trajectory planning.. Curr Op Neurobiol.

[5] Karniel A, Inbar GF (2000). Human motor control: learning to control a time-varying non-linear many-to-one system.. IEEE Transactions on systems, man and cybernetics.

[6] Mussa-Ivaldi FA, Bizzi E (2000). Motor learning through the combination of primitives.. Philos Trans R Soc London.

[7] Wolpert DM, Ghahramani Z (2000). Computational principles of movement neuroscience.. Nature Neurosci.

[8] Karniel A, Mussa-Ivaldi FA (2003). Sequence, time or state representation: how does the motor control system adapt to variable environments?. Biol Cybernetics.

[9] Shadmehr R, Mussa-Ivaldi FA (1994). Adaptive representation of dynamics during learning of a motor task.. Journal of Neuroscience.

[10] Pressman A, Welty LH, Karniel A, Mussa-Ivaldi FA (2007). Perception of delayed stiffness.. The International Journal of Robotics Research (IJRR).

[11] Nisky I, Baraduc P, Karniel A (2010). Proximodistal Gradient in the Perception of Delayed Stiffness.. Journal of Neurophysiology.

[12] Miall RC, Foulkes AJM (2000). Adaptation to visual feedback delays in a human manual tracking task.. Exp Brain.

[13] Cunningham DW, Chatziastros A, Von Der Heyde M, Bulthoff HH (2001). Driving in the future: temporal visuomotor adaptation and generalization.. J Vision.

[14] Kitazawa S, Kohno T, Uka T (1995). Effects of Delayed Visual Information on the Rate and Amount of Prism Adaptation in the Human.. Journal of Neuroscience.

[15] Smith WM, Bowen KF (1980). The effects of delayed and displaced visual feedback on motor control.. J Mot Behav.

[16] Krakauer JW, Ghilardi MF, Ghez C (1999). Independent learning of internal models for kinematic and dynamic control of reaching.. Nat Neurosci.

[17] Rabe K, Livne O, Gizewski ER, Aurich V, Beck A (2009). Adaptation to visuomotor rotation and force field perturbation is correlated to different brain areas in patients with cerebellar degeneration.. Journal of Neurophysiology.

[18] Thoroughman KA, Shadmehr R (2000). Learning of action through adaptive combination of motor primitives.. Nature.

[19] Padoa-Schioppa C, Li CSR, Bizzi E (2004). Neuronal activity in the supplementary motor area of monkeys adapting to a new dynamic environment.. Journal of Neurophysiology.

[20] Inbar GF, Yafe A, Homma S (1976). Parameter and Signal Adaptation in the Stretch Reflex Loop.. Progress in Brain Research.

[21] Gribble PL, Ostry DJ (2000). Compensation for loads during arm movements using equilibrium-point control.. Experimental Brain Research.

[22] Conditt MA, Mussa-Ivaldi FA (1999). Central representation of time during motor learning.. Proceedings of the National Academy of Sciences of the United States of America.

